# The frequency of missed breast cancers in women participating in a high-risk MRI screening program

**DOI:** 10.1007/s10549-018-4688-z

**Published:** 2018-01-31

**Authors:** S. Vreemann, A. Gubern-Merida, S. Lardenoije, P. Bult, N. Karssemeijer, K. Pinker, R. M. Mann

**Affiliations:** 1 Department of Radiology and Nuclear Medicine, Radboud University Medical Center, Geert Grooteplein 10, 6525 GA Nijmegen, The Netherlands; 2 Department of Pathology, Radboud University Medical Center, Nijmegen, The Netherlands; 3 Division of Biomedical Imaging and Image-guided Therapy, Division of Molecular and Gender Imaging, Medical University Vienna, Vienna, Austria; 4 Molecular Imaging and Therapy Service, Department of Radiology, Memorial Sloan Kettering Cancer Center, New York, USA

**Keywords:** Breast cancer, Breast MRI, High-risk screening, Prior MRI scan, Visibility

## Abstract

**Purpose:**

To evaluate the frequency of missed cancers on breast MRI in women participating in a high-risk screening program.

**Methods:**

Patient files from women who participated in an increased risk mammography and MRI screening program (2003–2014) were coupled to the Dutch National Cancer Registry. For each cancer detected, we determined whether an MRI scan was available (0–24 months before cancer detection), which was reported to be negative. These negative MRI scans were in consensus re-evaluated by two dedicated breast radiologists, with knowledge of the cancer location. Cancers were scored as invisible, minimal sign, or visible. Additionally, BI-RADS scores, background parenchymal enhancement, and image quality (IQ; perfect, sufficient, bad) were determined. Results were stratified by detection mode (mammography, MRI, interval cancers, or cancers in prophylactic mastectomies) and patient characteristics (presence of *BRCA* mutation, age, menopausal state).

**Results:**

Negative prior MRI scans were available for 131 breast cancers. Overall 31% of cancers were visible at the initially negative MRI scan and 34% of cancers showed a minimal sign. The presence of a *BRCA* mutation strongly reduced the likelihood of visible findings in the last negative MRI (19 vs. 46%, *P* < 0.001). Less than perfect IQ increased the likelihood of visible findings and minimal signs in the negative MRI (*P* = 0.021).

**Conclusion:**

This study shows that almost one-third of cancers detected in a high-risk screening program are already visible at the last negative MRI scan, and even more in women without *BRCA* mutations. Regular auditing and double reading for breast MRI screening is warranted.

## Introduction

Breast magnetic resonance imaging (MRI) is recognized as the most sensitive imaging method for the early detection of breast cancer [1]. Therefore, women at a lifetime risk for breast cancer development of ≥ 20% are invited for intensified screening programs including both mammography and breast MRI. Recent prospective studies have reported a sensitivity of MRI of around 90% using these screening settings [1–5].

Studies investigating the performance of mammography screening programs have consistently shown that between 31 and 50% of cancers detected in follow-up could have been detected at an earlier screening round [6–9]. This is one reason for the implementation of double reading in mammography screening [10, 11]. Furthermore, the frequency of these errors in mammography screening is nowadays regularly audited.

Although recently auditing guidelines for breast MRI screening were published by the American college of radiology [12], these do not include the evaluation of prior screening rounds in women who present with breast cancer. A few studies that focused on reasons and features of missed cancers in MRI screening showed that between 47 and 56% of cancers could have been detected at an earlier MRI examination [13, 14]. However, to our knowledge, no studies report on the visibility of interval cancers, tumors detected on mammography, or tumors in prophylactic mastectomy specimen in the prior MRI scans.

Therefore, the purpose is to evaluate the frequency of visible breast cancers detected in women participating in a high-risk screening program, that were missed on a prior MRI scan (screen-detected, interval, and cancers in prophylactic mastectomy specimen), and to assess patient and imaging factors contributing to non-detection.

## Materials and methods

### Ethics

The local institutional review board approved this study and the requirement for informed consent was waived.

### Breast cancer screening in women at intermediate-to-high risk for breast cancer

The breast cancer screening program for women at high or intermediate risk (≥ 20–25% lifetime risk) at our institution consists of annual breast MRI and mammography, usually at the same day, although some women opt for having the examinations 6 months apart. In women with germline *BRCA* mutations, screening starts from the age of 25 years, and the first 5 years no mammography is performed. In other women at increased risk for breast cancer, screening starts from the age of 35 or 45.

All women underwent contrast-enhanced breast MRI in prone position using a dedicated breast coil on a 1.5- or 3-T MRI scanner (Siemens, Erlangen, Germany). Contrast (Gd-DOTA, Guerbet, France) was administered through an iv-canula in the cubital vein at a dose of 0.1 mmol/kg using a power injector (Warrendale, Medrad, PA). Acquisition parameters changed over time, and are reported in detail elsewhere [15]. All MRI scans contained a 3D T1-weighted sequence that was performed before and 4–5 times after contrast administration. Clinical reporting was done by one of eight board-certified breast radiologists with between 6 months and 23 years of experience in breast MRI using a dedicated breast MRI workstation (versions of DynaCAD, Philips, Best, the Netherlands), that automatically yields subtraction images, maximum intensity projections (MIPs), and enhancement curves. All mammograms were obtained on full-field digital mammography machines (Senograph DS, Senograph 2000, GE, USA) by dedicated mammography technicians. Mammography was always performed in two planes (medio-lateral oblique and cranio-caudal), with optional further acquisitions. Clinical reporting using dedicated mammography monitors was done by the radiologists who also reported the MRI scan.

### Case selection

All screening breast MRI and mammography exams performed from 01 January 2003 to 01 January 2014 were identified by a cross-computer search. This yielded 9571 screening MRI studies and 6553 mammograms obtained in 2773 women. This database was linked to the nationwide population-based Netherlands Cancer Registry. In our cohort, 164 women presented with a total of 179 cancers. For these women, we noted age, menopausal status, and the reason for intensified screening: *BRCA1*, *BRCA2*, family history of breast cancer, personal history of breast cancer, or other (including germline PTEN mutation, previous radiation to the chest, hormone replacement therapy, and lobular carcinoma in situ in an earlier biopsy).

Cancers (invasive cancers or ductal carcinoma in situ (DCIS)) were subsequently categorized into MRI-detected, mammography-detected, interval cancers, and cancers detected in prophylactic mastectomy specimen (further referred to as incidental cancers). For each of these categories, we obtained the images acquired at the time of tumor detection and the last MRI reported to be negative. The time between cancer diagnosis and the last negative MRI scan was recorded. When no prior negative MRI was available, the case was excluded (*N* = 48).

#### MRI-detected cancers

Cancers were considered MRI-detected when they were screen-detected and mentioned in the MRI report at the time of diagnosis (hereafter called the current MRI). The most recent breast MRI scan reported to be negative between 6 and 24 months before cancer diagnosis was selected for re-evaluation.

#### Mammography-detected cancers

Cancers were considered mammography-detected when they were screen-detected and described in the mammography report, but the MRI report was negative. In this case, the MRI performed within the same screening round as the mammogram was re-evaluated, and therefore, the time between detection and the last negative MRI scan is negligible.

#### Interval cancers

Interval cancers were defined as cancers that were detected in between screening rounds due to symptoms. The last MRI prior to the cancer detection was selected.

#### Incidental cancers

Incidental cancers were defined as cancers detected in prophylactic mastectomy specimens with negative prior imaging. We selected the last MRI scan prior to the prophylactic mastectomy for assessment.

### Retrospective MRI interpretation

The last negative breast MR images were re-evaluated in consensus by two breast radiologists with respectively 8 and 12 years of experience in breast MRI. Readers were informed of the cancer location on the positive MRI and/or the histopathology results. In the case of mammography-detected cancers, location described in the mammography report and histopathology results were given.

The review was performed on an in-house developed dedicated breast MRI workstation [16]. The workstation performed motion correction [17], and showed T1-weighted images, subtraction images, and MIPs for all time points. The average contrast enhancement versus time curve was shown for the pointer location. For the MRI-detected cases, the current MRI was displayed alongside the prior MRI.

The readers, in consensus, scored whether the cancer was either invisible, if there was a minimal sign, or the cancer was visible in the last negative MRI in analogy to the Dutch auditing practice for mammography screening [7]. When the MRI was truly negative, the cancer was rated as invisible. Minimal signs were visible lesions at the site of the later detected cancer that, according to the consensus reading, would not likely be recalled in screening practice. Visible lesions were lesions that were present at the site of the later detected cancer and should have been recalled according to the consensus reading. All lesions in the current MRI, as well as all lesions visible or showing a minimal sign in the prior MRI, were assessed according to the Breast Imaging Reporting and Data (BI-RADS) MR-lexicon and BI-RADS scores were given accordingly [12, 18].

For each MRI scan, background parenchymal enhancement (BPE) was scored as minimal, mild, moderate, or marked. In addition, image quality (IQ) was subjectively scored as perfect, sufficient, or bad.

### Performance measures

We first assessed the frequency of visible findings and minimal signs in the negative MRI scans, overall and in the subgroups (MRI-detected, mammography-detected, interval cancers, and incidental cancers). Subsequently, we investigated whether patient factors (age at cancer detection, menopausal state, presence of a *BRCA* mutation) and imaging factors (field strength of MRI scanner, BPE, reported IQ) were related to the likelihood of missed lesions in prior breast MRI scans. For statistical analysis, we used Pearson’s Chi square tests for categorical variables and one-way ANOVA for continuous variables. The Tukey post-hoc test was used to compare the differences between the groups in case of continuous variables. To assess correlations, Spearman’s rho was computed for ordinal values and when data were not normally distributed (time between scan and cancer detection). A two-sided *P* value of ≤ 0.05 was considered statistically significant. All statistics were performed in SPSS (v22, SPSS Inc., Chicago, IL).

## Results

Between 01 January 2003 and 01 January 2014, 131 breast cancers were detected in women participating in the intermediate- and high-risk screening program, for whom a prior (or in case of mammography-detected cancers current) negative MRI scan was available (Table 1). Of these cancers, 76 were MRI-detected, 13 mammography-detected, 16 were interval cancers, and 26 were incidental cancers.

**Table 1:** Time to negative MRI and time to cancer diagnosis (in months), stratified by detection mode and BI-RADS scores of prior MRI scans

	MRI detect	MG detect	Interval^a^	Incidental^b^	Overall
*N* (%)	76 (58)	13 (10)	16 (12)	26 (20)	131
Mean patient age in years (sd)	49.5 (11.2)	53.4 (8.3)	41.3 (9.1)	42.5 (10.6)	47.5 (11.2)
Time to last negative MRI in months (sd)	11.5 (3.0)	1.2 (3.4)	8.6 (3.1)	2.8 (3.1)	9.5 (4.5)
Histology					
IDC	49 (70)	2 (3)	15 (21)	4 (6)	70
ILC	4 (67)	0 (0)	1 (17)	1 (17)	6
DCIS	13 (33)	8 (21)	0 (0)	18 (46)	39
Mixed IDC and ILC	7 (78)	0 (0)	0 (0)	2 (22)	9
Other	3 (43)	3 (43)	0 (0)	1 (14)	7
Tumor stage at detection					
pTis	13 (33)	8 (21)	0 (0)	18 (46)	39
pT1mic	0 (0)	0 (0)	0 (0)	1 (100)	1
pT1a/b/c	7/22/18 (72)	3/1/0 (6)	0/1/7 (12)	2/3/1 (9)	12/27/26
pT2	12 (71)	1 (6)	3 (18)	1 (6)	17
pT3	1 (100)	0 (0)	0 (0)	0 (0)	1
pT4D	1 (100)	0 (0)	0 (0)	0 (0)	1
Recurrence	1 (33)	0 (0)	2 (67)	0 (0)	3
Unknown	1 (25)	0 (0)	3 (75)	0 (0)	4
Nodal status at detection					
pN0	52 (60)	11 (13)	7 (8)	17 (20)	87
pN+	16 (67)	1 (4)	5 (21)	2 (8)	24
Unknown	8 (40)	1 (1)	4 (20)	7 (35)	20
Visibility on prior					
Invisible	21 (47)	6 (13)	8 (18)	10 (22)	45
Minimal sign	31 (69)	4 (9)	3 (7)	7 (16)	45
Visible	24 (59)	3 (7)	5 (12)	9 (22)	41

In between parenthesis, the percentage of lesions in the specified category is given, except when indicated otherwise

^a^Interval cancers were defined as cancers detected in between screening rounds

^b^Incidental cancers were defined as cancers detected in prophylactic mastectomy specimens

**Fig. 1:**
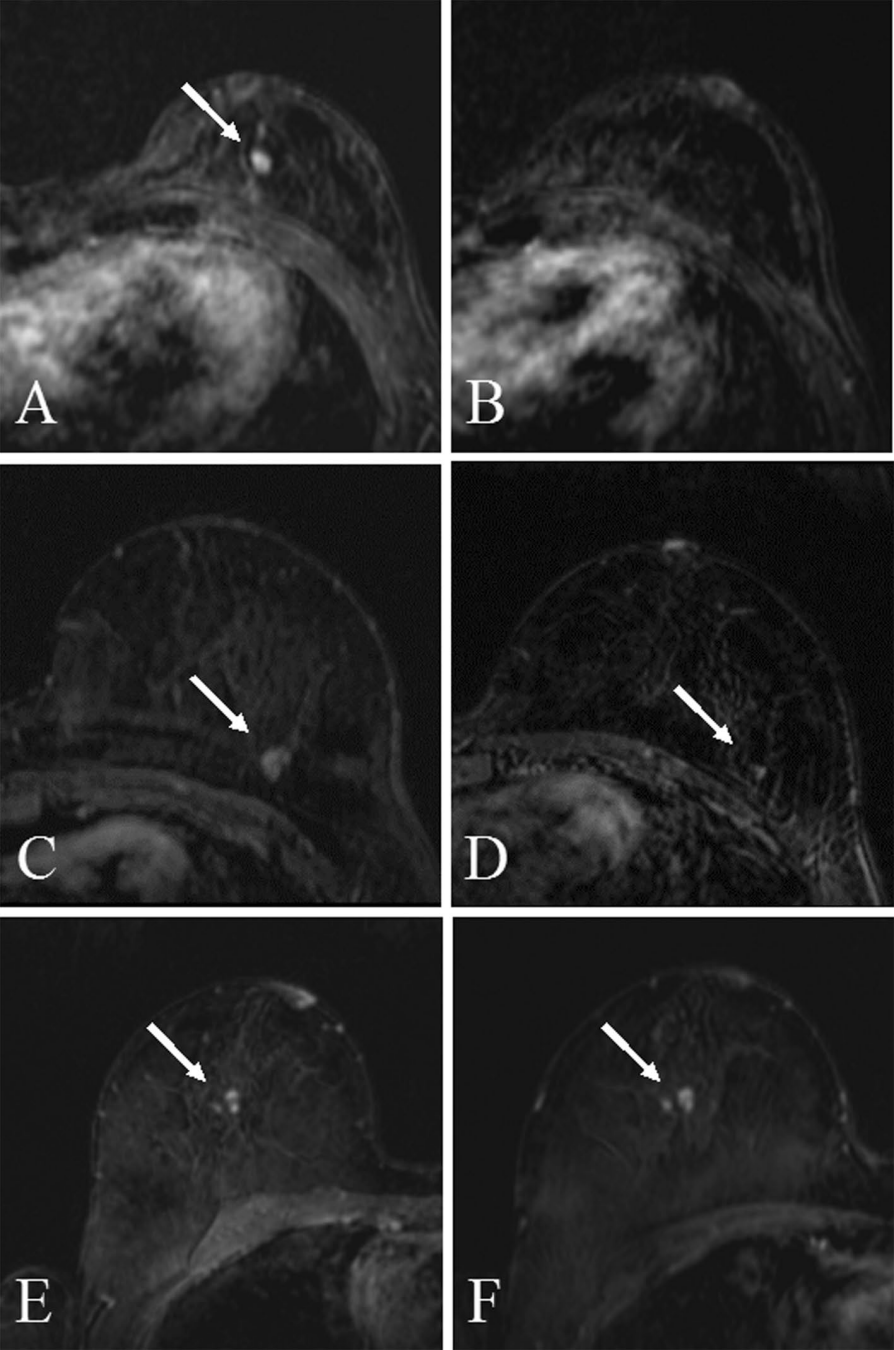
First the subtraction images of a breast cancer invasive ductal carcinoma (IDC) grade 3 (**a**), which were rated as invisible in the prior MRI (**b**), second row are the images (**c**) rated as minimal sign in the prior image (**d**) also showing an IDC grade 3, and the last row are the images of the current MRI (**e**) and the visible lesion in the prior MRI (**f**) an invasive lobular carcinoma grade 2

In the 131 re-evaluated MRI scans, lesions were considered invisible in 45 cases (34%). A minimal sign was present in 45 cases (34%) and lesions were visible in 41 cases (31%). Figure 1 presents examples of lesions in the three visibility categories. Of all visible lesions, 2 (5%) were re-evaluated as BI-RADS-3, 35 (85%) as BI-RADS-4, and 4 (10%) as BI-RADS-5. Lesions that showed a minimal sign were scored as BI-RADS-2 in 22 cases (49%), and BI-RADS-3 in 23 cases (51%). Overall, 64 cases (49%) of prior negative MRI scans were scored as BI-RADS-3 or higher.

Table 2 shows the distribution of these findings in each subgroup. We did not observe a significant difference based upon the mode of cancer detection (*P* = 0.447). However, the frequency of visible findings in negative MRI scans of women with mammography-detected cancers was somewhat lower than in other categories, which is likely related to the simultaneous interpretation of mammograms and MRI scans in these cases and the fact that 8 of 13 cancers in this category were pure DCIS. It should be noted that 5 of 16 (31%) interval cancers were already visible at the last scan, and were all scored as BI-RADS-4 or 5.

**Table 2:** Visibility of lesions on prior negative MRI scans, stratified by patient and imaging factors

	*N* (%)	Invisible^a^ (%)	Minimal sign^a^ (%)	Visible^a^ (%)	*P* value^b^
Overall	131 (100)	45 (34)	45 (34)	41 (31)	N/A
Patient factors					
Mean age at cancer detection in years (sd)	47 (11.3)	44 (11.4)	46 (9.8)	52 (11.4)	0.003
Menopausal status					0.229
Premenopausal	60 (46)	25 (42)	17 (28)	18 (30)	
Postmenopausal	71 (54)	20 (28)	28 (39)	23 (32)	
Reason for screening					0.001
BRCA^c^	70 (53)	34 (49)	23 (33)	13 (19)	
Non-BRCA	61 (47)	11 (18)	22 (36)	28 (46)	
Imaging factors					
Field strength					0.895
1.5 T	96 (73)	33 (34)	32 (33)	31 (32)	
3 T	35 (27)	12 (34)	13 (37)	10 (29)	
BPE					0.570
Minimal	83 (63)	30 (36)	27 (33)	26 (31)	
Mild	22 (17)	5 (23)	9 (41)	8 (36)	
Moderate	12 (9)	3 (25)	4 (33)	5 (42)	
Marked	14 (11)	7 (50)	5 (36)	2 (14)	
Reported IQ					0.021
Perfect	111 (85)	43 (39)	34 (31)	34 (31)	
Sufficient	18 (14)	1 (6)	10 (56)	7 (39)	
Bad	2 (2)	1 (50)	1 (50)	0 (0)	

In between parenthesis, the percentage of lesions in the specified category is given, except when indicated otherwise

^a^According to the two readers in consensus in the prior MRI scan

^b^*P* value is based on Pearson Chi square test evaluating the differences between the three visibility categories (invisible, minimal sign, and visible)

^c^The *BRCA* population included also untested first-degree relatives

*IQ* image quality

Table 2 also shows the impact of patient and imaging factors on the likelihood of false-negative MRI reports. Overall, the frequency of missed findings is influenced by age, the reason for screening, and reported image quality. Especially the presence of a *BRCA* mutation strongly reduces the frequency of visible findings in prior negative MRI scans.

## Discussion

Our results show that, of 131 breast cancers with negative prior MRI exams retrospectively evaluated in this study, 31% were already visible on this negative exam. In fact, 29% of the 131 lesions were rated as BI-RADS-4 or BI-RADS-5 and should thus have been recalled based on these prior exams. When including cancers presenting with minimal signs, 65% of the lesions were already recognizable on the prior MRI exam. Both from a learning perspective and in terms of liability, it is essential that these figures are available.

These results are in line with reports from Yamaguchi et al. [13], and Pages et al. [14] who, in smaller cohorts (15 and 58 patients), reported that 56 and 47% of breast cancers were already visible on prior MRI examinations and retrospectively assessed as BI-RADS-3 or higher. Both studies, however, only included screen-detected cancers on MRI. To our knowledge, this study is the first to show that also 31% of interval cancers and even 35% of incidental cancers can be identified at the last negative MRI scan.

The program sensitivity of our high-risk screening cohort was, as reported earlier, 89.7% [19], which is comparable to recent prospective studies on MRI screening in women at increased risk such as the Italian Hibcrit trial (91%) [3] and the German EVA trial (93%) [4]. Therefore, it is likely that our findings are applicable to all breast MRI screening settings. Our findings related to visibility of cancers in prior examinations are also similar to those found in mammography screening. Previous studies [7] have reported that up to 50% of the cancers detected in mammography screening with double reading were already visible at earlier screening examinations and approximately half of these were suspicious.

The clinical consequences of missing a cancer in an MRI scan obtained before prophylactic mastectomy are relatively minor. Most of these cancers are DCIS only. This is a relative frequent finding in prophylactic mastectomy specimen (approximately 5% in our institution) [20]. Since the period between the MRI scan and the subsequent prophylactic mastectomy is usually short, it is unlikely that missing these lesions will alter the patients’ prognosis. Nonetheless, the psychological impact might be substantial and should be taken into account [21].

The consequences of missing breast cancers that present subsequently as interval cancer, on the other hand, are dire. These cancers usually are invasive, poorly differentiated and fast growing [22]. Detection of these cancers at the MRI examinations could still have had a significant effect on subsequent prognosis and warrants investigation of methods to reduce false-negative reporting of MRI scans.

In the group of mammography-detected cancers, the number of visible cancers is somewhat lower than in the other groups, though this did not reach statistical significance. This result was expected as the examinations are in practice often reported simultaneously. The fact that we did find four cancers on the current MRI scan that were re-evaluated as visible in retrospect can therefore only be explained by underreporting of subtle findings on the MRI scan due to already significant findings on the mammogram that would warrant a biopsy anyway. In addition, of note is that of four invasive cancers in this mammography category only one cancer was also regarded as invisible in the consensus reading. The finding that the frequency of invisible in situ cancers is substantial (50%) underlines that, although the sensitivity of breast MRI is high, it is not 100%, as we are certain these cancers are present in the breast.

**Fig. 2:**
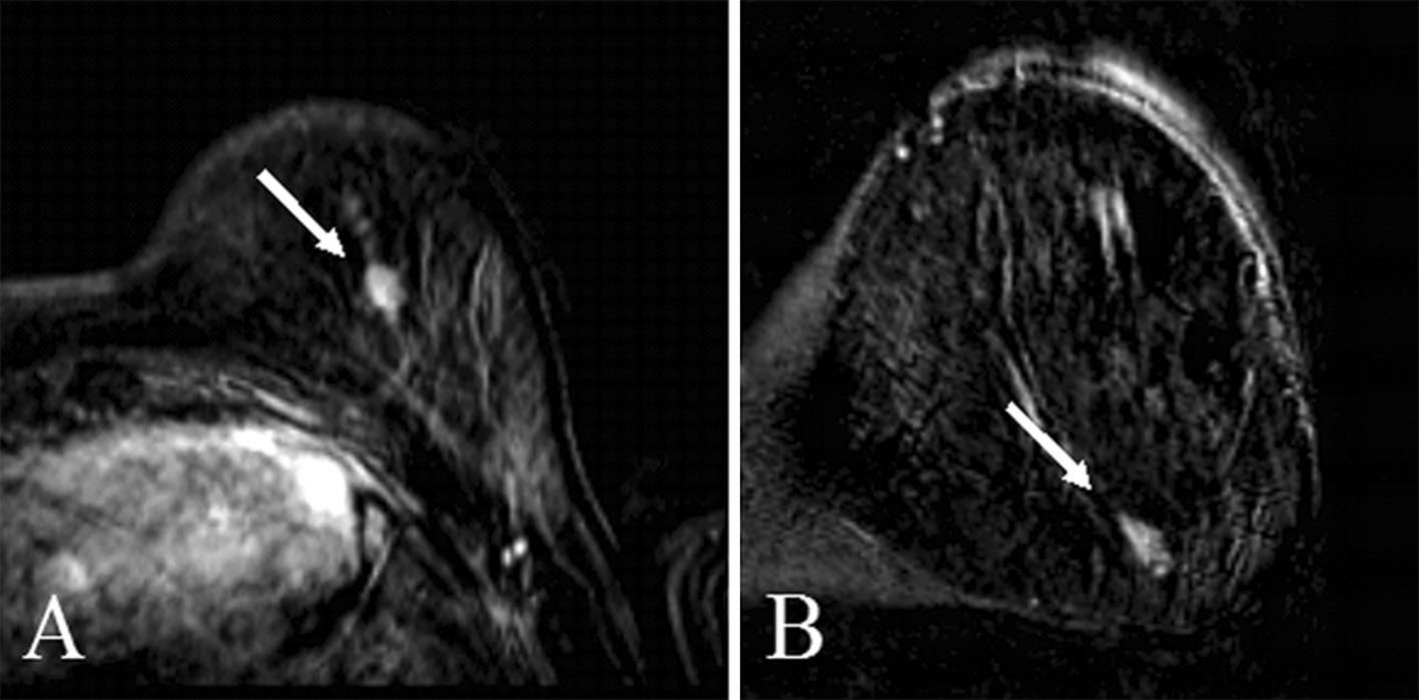
An example of an interval cancer detected on ultrasound 9 months after prior MRI. This visible lesions was scored as BI-RADS 5 in re-evaluation; **a** subtraction image of an invasive ductal carcinoma grade 2 (pT1cN1mi (sn)) in transversal plane and **b** in coronal plane

The fact that the frequency of visibility of late(r) detected cancers is virtually independent from the eventual mode of cancer detection implies that the problem should be sought in the evaluation of MRI scans itself. Radiologists in general may make two types of mistakes. The first is known as overlook error. The abnormality is simply not seen, and therefore cannot be classified correctly. The second is known as interpretation error. In this case, the lesion is seen but falsely interpreted as benign finding, and might therefore not even be reported, although the most evident cases of interpretation errors obviously mention the lesion as benign in the report [13, 14, 23, 24]. In the evaluation of screening breast MRI, likely both processes play a role. In retrospect, four cases were classified as BI-RADS-5. It is unlikely that these lesions were seen but not recalled (Fig. 2). It is far more likely that these cases were overlooked. However, the vast majority (*N* = 35) of visible cases were classified in the prior exam as BI-RADS-4. In these cases, interpretation errors might have prevented recall in clinical practice. This is also supported by the fact that the strongest modifier of the frequency of visible lesions is the presence of a *BRCA* mutation (49% of the lesions were truly invisible in *BRCA* mutation carriers compared to 18% in others). The much higher a priori chance of these women to develop breast cancer compared to other women at increased risk [25], along with the fast growth of cancers in these women [26], leads to lowering the threshold for recall. In other words, women with *BRCA* mutations are recalled for lesions that would have been ignored in women without *BRCA* mutations. This might also explain the slightly younger age of women with invisible lesions at the last negative MRI, as the *BRCA* population in general is younger than the non-*BRCA* population.

We also observed that IQ was associated to the visibility score, with more frequent visible findings in negative reported MRI scans of less than perfect quality. This implies that striving for excellent image quality is important. When a scan is of inferior quality, for example due to motion artifacts, rescanning the patient should be considered. We did not detect an effect of field strength on the visibility of lesions in prior negative MRI scans, and therefore underline that adequate MRI scans can be performed at both 1.5- and 3-T systems. BPE was not statistically related to the likelihood of visibility of lesions in the last negative MRI scan. Nevertheless, in women with marked BPE the frequency of visible lesions was with 14% substantially lower than in other groups. We assume that very strong BPE might in fact sometimes obscure lesions that would otherwise be detected [27], though larger numbers are required for statistical analyses.

While an obvious solution to reduce misinterpretation of eventual cancers would be to lower the threshold for recall in non-*BRCA* mutation carriers, this cannot be straightforwardly applied. We earlier analyzed the positive predictive value of biopsy (PPV3) in women in this cohort [19], which were 0.38 and 0.29 for *BRCA1* and *BRCA2* carriers, respectively. However, in women with a positive family history but no *BRCA* mutation PPV3 was only 0.14, which implies that lowering the threshold for recall in these women might lead to unacceptable high biopsy rates for benign lesions, and might further jeopardize the cost-effectiveness of MRI screening. The optimal balance between recall and cancer detection for women in different risk categories has yet to be determined.

We acknowledge that in the consensus read a bias was introduced by the non-blinded fashion of the evaluation. Readers were aware of the breast cancer location on the current MRI scan, mammography, or in the excised specimen. While this is common practice in auditing, it has been postulated that this approach is too harsh and leads to more lesions classified as visible than what would have been the case in a blinded setting where cases are mixed with normal scans [28]. However, in a previous study by Gubern-Merida et al. [29], a CAD system was used for the detection of breast cancer in a subset of 40 of the cancer cases that were used in the current evaluation, mixed with 120 normal scans. At four false-positives per normal scan, the sensitivity of the CAD system was 0.71 and 0.31 for visible and minimally visible lesions in the prior negative scans, respectively, whereas sensitivity was 0.82 for the respective current scans in which the cancers were actually diagnosed. This implies that indeed a substantial subset of the cancers is at least detectable in the prior negative scans and patients could therefore indeed benefit from CAD-assisted reading or a blinded second read.

This study has some other limitations. It is a single-center study based upon retrospective analysis of screening data. However, the fact that the program sensitivity is in line with published prospective trials implies that the findings are likely generalizable to similar bi-modal screening programs for women at increased risk. In addition, the prospective reads of the scans were conducted by one of eight radiologists with a strong variability (6 months to 23 years) in experience with breast MRI, which also reflects clinical practice. The numbers of missed cancers are too small to assess the effect of experience on false-negative reporting. MRI protocols changed over time. However, all examinations were performed with at the time state-of-the-art equipment and imaging quality was in adherence with international recommendations [30, 31]. Our numbers are likewise too small to detect whether MRI protocol changes might have influenced the frequency of visible findings in negative MRI scans over time.

In conclusion, our results show that almost one-third of breast cancers can be detected at the last reported negative MRI. Since this is true for both screen-detected and interval cancers, it is essential to find methods in order to reduce reading errors. As a first step, regular auditing of clinical practice seems to be indicated. In addition, structural double reading of breast MRI exams may be of value, although further research on the added cancer detection yield is required. Computer-aided detection tools for cancer detection in breast MRI might play a significant role in the prevention of reading errors in the future.
